# Comprehensive conservative treatment for multiple metastases of skull osteosarcoma: A case report

**DOI:** 10.3389/fneur.2023.1037673

**Published:** 2023-01-26

**Authors:** Dawei Jiang, Jingming Wang, Qian Chen, Junyi Wu, Ming Xu, Xiuchun Yu

**Affiliations:** Department of Orthopedics, 960th Hospital of PLA, Jinan, China

**Keywords:** skull osteosarcoma, bone metastases, pulmonary metastasis, Denosumab, Anlotinib

## Abstract

**Background:**

Skull osteosarcoma is relatively rare, and it is difficult to be diagnosed according to medical history and imaging examination due to the complex structure and diverse components of the brain. Consequently, there is only a limited number of patients who can undergo neoadjuvant chemotherapy before the operation. Although neoadjuvant chemotherapy plays an important role in the treatment of osteosarcoma, there is still a “bottleneck” in the current treatment method which when pulmonary metastasis occurs, or surgical treatment is not Enneking appropriate. Under such circumstances, the choice of treatment can be an issue.

**Case:**

A 16-year-old male patient with multiple metastases of skull osteosarcoma was reported. The patient suffered not only tinnitus and hearing loss in the right ear but also right facial paralysis and headache. The preoperative brain MRI showed a tumor in the right cerebellopontine angle (CPA) area. He underwent skull tumor resection at another hospital in November 2018, during which process the biopsy revealed epithelioid osteoblastoma-like osteosarcoma. The patient had supplemental radiotherapy 1 month after surgery because of tumor recurrence. 32 months afterward, pulmonary metastases and multiple bone metastases were found. Then the patient underwent multiple conservative treatments which include Denosumab, Anlotinib, and DIA (cisplatin + ifosfamide + doxorubicin) chemotherapy at our hospital. After a series of 6 cycles of treatment, the patient can walk without aid. Lactate dehydrogenase (LDH) and Alkaline phosphatase (AKP) returned to a normal level. Fluorodeoxyglucose (FDG) metabolism in all bone metastases decreased to normal except for the ones in the proximal left femur, and the FDG metabolism in the left femur is significantly lower than that before treatment. Multiple bone metastases showed different extents of high-density calcification, and the volume of the local bone metastases has been reduced significantly. The patient‘s condition stayed stable at latest follow-up.

**Conclusion:**

We found that multiple conservative treatments, which include Denosumab, Anlotinib and DIA chemotherapy, can improve patients' life quality, and help avoid further osteolytic destruction for patients with skull osteosarcoma and multiple metastases. Its specific mechanism and scope of the application still need to be further studied.

## Introduction

Primary skull osteosarcoma is relatively rare, only accounting for about 1.6% of osteosarcoma in general and 1–2% of skull tumors (1). It can originate from the extracranial tissues, such as the mesenchyme of the dura, subarachnoid cistern, and perivascular sheath, and it can also evolve from intracranial fibrosarcoma and skull osteoma. Primary skull osteosarcoma mainly occurs in teenagers and people in their 50 s, without obvious gender differences. According to the anatomical site of the tumor, it can be manifested as headache, nausea, tinnitus, cranial nerve palsy, exophthalmos, and visual impairment (2).

Skull osteosarcoma was reported for the first time by Garland in 1945, and a total of 321 cases have been found since then (1). Because of its special location, 14.1% of skull osteosarcoma have intracranial involvement, so it is difficult to remove the tumor completely (3). The overall probability of recurrence and metastasis is high, and the prognosis is poor. The 5-year survival rate is about 50%, the median survival period is 33 months, and the median survival period without progression is 12 months. Once bone metastasis occurs, the 5-year survival rate decreases to about 13% (4–6).

Due to the rare incidence of skull osteosarcoma with multiple metastases, the standard therapy has not yet formulated. We treated 1 patient with multiple metastases of skull osteosarcoma in our department. After a series of comprehensive conservative treatments including Denosumab, Anlotinib, and DIA (cisplatin + ifosfamide + doxorubicin) chemotherapy, the patient was pain-free and could walk without aid. The osteolytic destruction region resulting from bone metastases showed obvious calcification, the SUV value of multiple metastases decreased, and the volume of local bone metastases was reduced significantly. The clinical outcome was satisfactory. There is no report on the treatment of lung and multiple bone metastases of skull osteosarcoma with Denosumab, Anlotinib, and DIA chemotherapy in the literature. The special report is as follows.

## Case report

**Figure 1:**
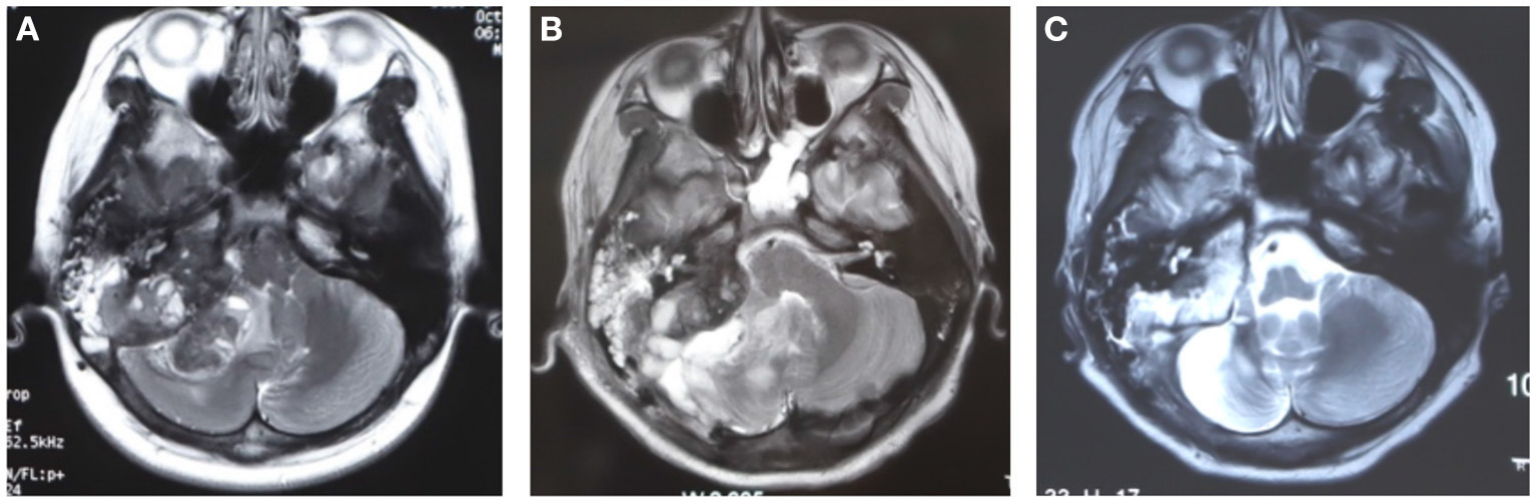
The brain MRI images before and after primary surgery. **(A)** The preoperative image showed a tumor occupied in the posterior fossa, medial to the right mastoid bone. **(B)** The postoperative 9 days MRI after surgery showed edema in the surgical region, some residual tumor existed for being unable to resect the tumor completely. **(C)** The MRI image showed that the tumor remained stable 6 months after radiotherapy.

A 16-year-old male was hospitalized in our department in September 2021 because of a month-long history of low back pain and left hip pain. The patient underwent tumor resection in the right cerebellopontine angle (CPA) area due to tinnitus in the right ear with hearing loss, right facial paralysis, and paroxysmal headache 33 months ago at another hospital. The preoperative brain MRI showed a tumor in the right cerebellopontine angle (CPA) area (Figure 1). A large tumor was seen in the condylar fossa and under the mastoid process, and the mastoid bone was destroyed by the tumor. After surgery, it was pathologically characterized as epithelioid osteoblastoma-like osteosarcoma. Although some residual tumor was found in postoperative MRI, the patient did not undergo further chemotherapy because his symptoms were relieved after surgery. The osteosarcoma in the right CPA area recurred in December 2018. Then the patient had postoperative radiotherapy (dt54gy/27F) to supplement the surgery. The patient had MRI examination 4, and 6 months after the radiotherapy, respectively. MRI indicated that the tumor stayed stable. Then because of the widespread of a novel coronavirus in China, the patient was not followed consistently every 3 months and did not have routine radiological examinations. The patient had low back pain and left hip pain in August 2021. X-ray images showed osteolytic destruction in the left femoral neck, and MRI images showed abnormal signals of T12, L2–L4, and S2 vertebral bodies, and related accessories, which was consistent with the manifestation of multiple vertebral body malignant tumors.

The patient was not able to walk at that time. Lactate dehydrogenase (LDH) was 271 u/L and alkaline phosphatase (AKP) was 2,166 u/L. PET-CT examination showed that the metabolism of fluorodeoxyglucose (FDG) increased in the lymph nodes near the acromion of the bilateral clavicle, the left 5th, and 6th posterior ribs and multiple parts of the whole body (T2–T12, L1–L4 vertebral bodies, T2, T6, T12 and L2 attachments, left 4th rib, right 11th posterior rib and 8th anterior rib, left clavicle, sacrum, femoral head and upper segment of the femur, right acetabulum, and ischium). High-density calcification was found at the left 5th and 6th posterior ribs, and osteolytic destruction was found in other regions. The biopsy of the left femur revealed osteosarcoma, and Ki-67 was about 30–40%.

This patient was diagnosed with skull osteosarcoma with multiple metastases. The metastases have been distributed in the lungs and multiple bones. According to the Enneking staging system, it is classified as stage 3, and further operation was not appropriate. Considering the osteolytic destructions caused by bone metastases, Denosumab (120 mg, once on the 1st, 8th, and 15th days of the first time, and once every month after that) was used. Meanwhile, according to the recommendations of CSCO classic osteosarcoma diagnosis and treatment guideline (version 2020), DIA neoadjuvant chemotherapy (cisplatin 120 mg/m^2^, 1 day; doxorubicin 30 mg/m^2^, 3 days; ifosfamide 2 g/m^2^, 5 days, 1 course for 2 weeks) were used. In addition, as the patient had pulmonary metastasis, Anlotinib (12 mg, once a day, 2, 1 weeks for rest, 1 course for 3 weeks) was also used. The patient and the patient's parents fully agree with the therapy.

**Figure 2:**
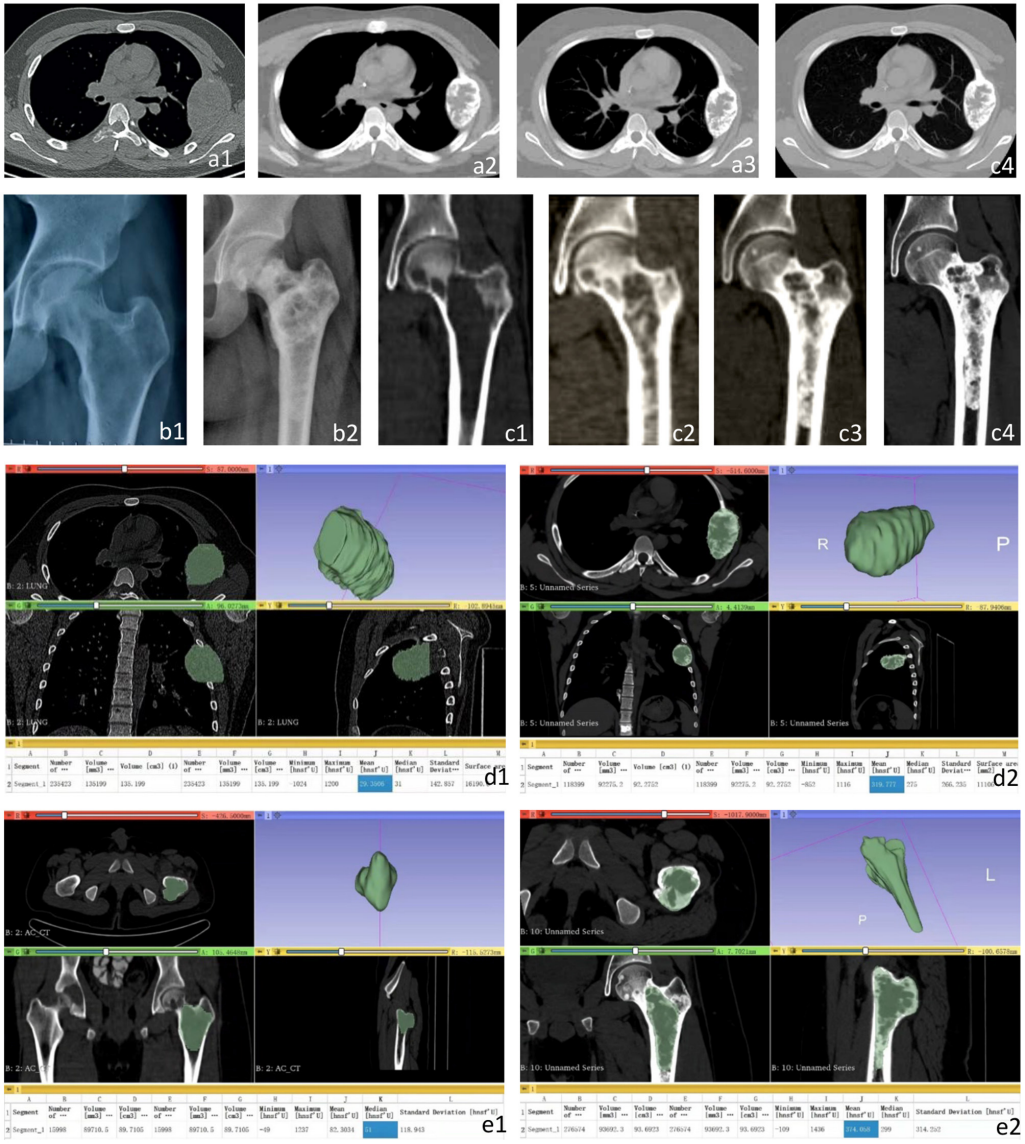
CT and X-ray images of the left fourth rib and proximal left femur before and after treatment. **(a1)** the left fourth rib showed expansive low-density changes before treatment; **(a2–a4)** A gradually expanding speckled high-density foci and sclerotic edges was detected in the left fourth rib before the 3^rd^ **(a2)**, 4^th^ **(a3)**, and 5^th^ **(a4)** chemotherapy, respectively. **(b1)** Osteolytic changes and local cortical thinning were found before treatment. **(b2)** After the 6th chemotherapy, X-ray images showed obvious calcification at the osteolytic destruction of the proximal left femur. **(c1)** Osteolytic changes at the proximal left femur and local cortical thinning were found in CT images before treatment. **(c2–c4)** The metastasis at the proximal left femur showed gradually expanding high-density foci and sclerotic edges before the 2^nd^ **(c2)**, 3^rd^ **(c3)**, and 4^th^ **(c4)** chemotherapy. Three-dimensional CT reconstruction of the left fourth costal metastasis and left proximal femoral metastasis before treatment and the fifth chemotherapy. **(d1)** the size of the metastasis before treatment was 135.20 cm^3^, and the average CT value was 29.35 hu. **(d2)** Before the 5th chemotherapy, the size of the metastasis was 92.28 cm^3^, and the average CT value was 319.78 hu. **(e1)** The size of metastases was 89.71 cm^3^, and the average CT value was 82.30 hu before treatment. **(e2)** The size of the metastasis was 93.69 cm^3^, and the average CT value was 374.06 hu before the 5th chemotherapy.

A total of 6 courses of DIA chemotherapy were completed, during which VAS of patients were regularly assessed, LDH and AKP were tested, X-rays and CT were rechecked (Figure 2). During the treatment, the patient developed myelosuppression, alopecia, nausea, and vomiting, but no other adverse events were found.

**Figure 3:**
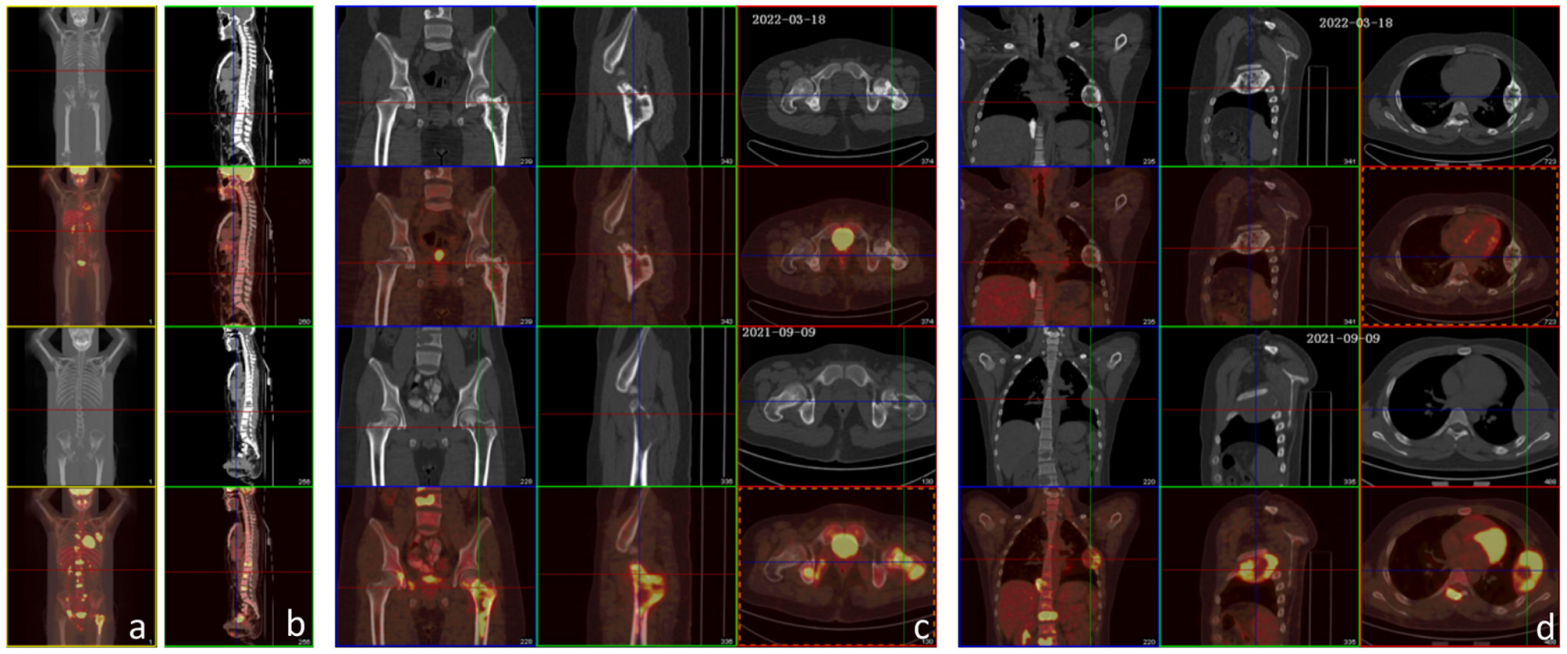
PET-CT examination before treatment and after the 6th chemotherapy. **(a–d)** The lower two rows are images before treatment, and the upper two rows are images after the 6th chemotherapy. After treatment, FDG metabolism in all metastases decreased (bilateral paraacromial side of the clavicle, left 5th and 6th posterior intercostal space, right lower lobe of the lung, lymph nodes in mesentery area, and multiple parts of the whole body: T2–T12, L1–L4 vertebral bodies, T2, T6, T12, and L2 attachments, left 4th rib, right 11th posterior rib and 8th anterior rib, left clavicle, sacrum, femoral head and upper segment of the femur, right acetabulum, and sciatic bone), except that the local FDG metabolism at the proximal left femur was still higher than normal (significantly lower than that before treatment). The multiple bone metastases showed varying extents of high-density calcification. There was little change from the mastoid process to the petrous tip of the right temporal bone, showing irregular morphology and increased bone density, and no significant increase in FDG metabolism.

**Figure 4:**
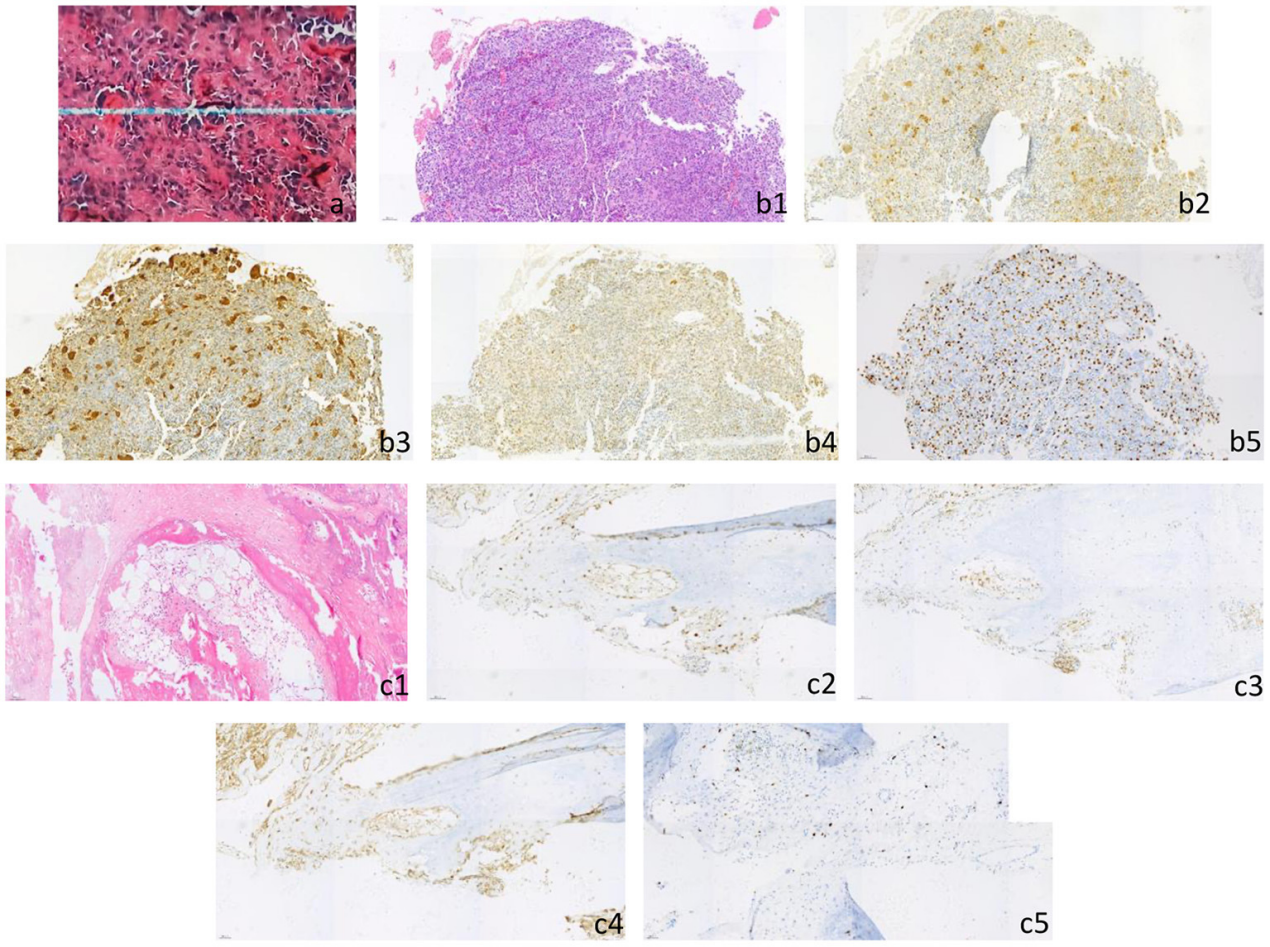
**(a)** Postoperative pathology of primary skull tumor showed that it was consistent with osteosarcoma (he × 200), immunohistochemical staining results: CD3 (scattered+), CD68 (scattered+), CD20 (occasionally+), lysozyme (occasionally+), CD34 (vascular+), PLAP (–), Ki-67 (focal about 20%). **(b1–b5)** The biopsy of left femur was pathologically consistent with osteosarcoma (he × 200), immunohistochemical staining results: CDK4 (+), CD68 (+), CD99 (+), MDM2 (+), h3k36m (+), SATB-2 (+), p53 (about 5%+), p16 (large cell+), NSE (focal+), H33g34w (–), p63 (–), S-100 (–), syn (–), Ki-67 (about 30–40% +). [**(b1)** HE, **(b2)** CDK4, **(b3)** CD68, **(b4)** MDM2, **(b5)** Ki-67]. **(c1–c5)** After the 6th chemotherapy, the pathology of left femur puncture biopsy was consistent with the pathological changes of skull osteosarcoma metastasis (he × 200), immunohistochemical staining results: SATB-2 (+), CD68 (focal+), CDK4 (partial+), p16 (partial+), p53 (small number of cells+), MDM2 (partial+), S-100 (–), H33g34w (–), h3k36m (–), p63 (–), Ki-67 (about 10%+). [**(c1)** HE, **(c2)** CDK4, **(c3)** CD68, **(c4)** MDM2, **(c5)** Ki-67].

After the conservative treatment, VAS decreased from 10 to 0, and LDH and AKP decreased to normal. PET-CT examination showed FDG metabolism in all the bone metastases decreased to normal, except that the local FDG metabolism in the proximal left femur was still high (significantly lower than that before treatment). The multiple bone metastases showed different extent of high-density calcification, and the volume of the metastases in the fourth left rib was reduced significantly (Figure 3). The left femoral puncture biopsy was performed again, and the pathology confirmed osteosarcoma metastasis, while Ki-67 decreased to about 10%+ (Figure 4).

After the last cycle of DIA chemotherapy in February 2022, the patient continued the treatment of Anlotinib and Denosumab. The patient followed consistently every 3 months. At the latest follow-up in November 2022, the patient‘s condition stayed stable.

## Discussion

For osteosarcoma patients with multiple lesions, whether the lesions were multiple primary tumors or metastatic disease from a dominant primary is still debatable. Multifocal osteosarcoma (MFOS) is characterized as a multicentricity of osseous osteosarcomas, either synchronous or metachronous, without visceral involvement (7). Some studies have shown several features of MFOS, including (1) absence of metastasis to the lung which rules out hematogenous spread, (2) equal response of predominant and secondary tumors following chemotherapy—which favors multiple synchronous primary lesions, and (3) without a history of Paget disease, metabolic bone disease or radiotherapy (8, 9). Otherwise, metastatic osteosarcoma was characterized as at least one lesion with features suggestive of primary osteosarcoma, with remaining lesions more suggestive of metastases. The metastatic lesions appear as purely sclerotic or heavily mineralized metaphyseal lesions with a narrow transition zone, no evidence of cortical destruction or soft tissue mass, or malignant periosteal new bone formation (7). Meanwhile, some studies conclude that MFOS represents one extreme of a vast spectrum of metastatic osteosarcoma (10, 11). This patient was diagnosed with multiple metastases of skull osteosarcoma for the following reasons: (1) Multiple bone metastasis was found 33 months after skull osteosarcoma, and the pathology confirmed the metastasis, (2) Lung metastasis was found when the patient was admitted in our hospital.

When skull osteosarcoma is diagnosed, it is better for the patient to undergo neoadjuvant chemotherapy, followed by resection of detectable disease and adjuvant chemotherapy, commonly in combination with adjuvant radiotherapy (12–14). Skull osteosarcoma is rare, only a total of 321 cases were found from 1973 to 2013 (1). It is difficult to be diagnosed using medical history and imaging examination due to the complex structure and diverse components of the brain. The pathological result is the gold standard of diagnosis. Consequently, there is a rare number of patients who can undergo neoadjuvant chemotherapy before the operation. In addition, it is difficult to resect the tumor completely, leading to a high postoperative recurrence and metastasis rate (1, 4). Radiotherapy is often used in the treatment of local recurrences, incomplete excision, and surgical treatment cannot be Enneking appropriate. However, it is not always successful due to the fact that skull osteosarcoma is relatively radiation-resistant (4, 5). Studies have shown that negative surgical margins and neoadjuvant chemotherapy are positively correlated with the prognosis of skull osteosarcoma (1–5, 15, 16). At present, the individualized combined use of doxorubicin, ifosfamide, cisplatin, and methotrexate has become the most effective first-line neoadjuvant chemotherapy for the treatment of osteosarcoma. A large number of studies have confirmed that neoadjuvant chemotherapy can inhibit tumor growth, and reduce tumor volume (15–17). It is reported in the literature that the application of neoadjuvant chemotherapy drugs has greatly increased the limb salvage surgery rate of osteosarcoma in China, and also increased the 5-year survival rate from < 20% to about 60% (16, 18). Although neoadjuvant chemotherapy plays an important role in the treatment of osteosarcoma, there is still a “bottleneck” in the current treatment of osteosarcoma. When pulmonary metastasis occurs, or surgical treatment is not Enneking appropriate, it would be difficult to make the right choice of appropriate treatment. With the continuous advances in molecular biology and immunology research, targeted drugs may have a significant impact on the treatment of osteosarcoma (18).

Denosumab is a human monoclonal antibody that interferes the bone remodeling process. It inhibits the interaction with RANK (expressed on the surface of osteoclasts and their precursors), by binding the receptor activator of the nuclear factor kappa-beta ligand (RANDKL). It mimics the activity of osteoprotegerin (OPG), thus inhibiting the activation of osteoclasts and delaying tumor progression (19). It is often used in clinical practice for bone metastases, multiple myeloma, and giant cell tumor of bone in solid tumors dominated by osteolytic destruction, and can also be used for secondary osteoporosis of tumors (20, 21). Studies have reported the expression of RANKL in osteosarcoma (22–25). Animal models of osteosarcoma have confirmed that RANKL blockade can prevent tumor progression, improve survival rate and inhibit pulmonary metastasis (26). RANKL expression among osteosarcoma patients was found to be related to poor response to chemotherapy, and a neoadjuvant chemotherapy combined with Denosumab can improve their survival rate (27). It has been reported in the literature that after two lines of chemotherapy and stereotactic radiotherapy for a patient with unresectable C7 and T1 vertebral osteosarcoma, the tumor progressed locally. After treatment with Denosumab and sorafenib, the tumor metabolism reached complete remission and lasted for more than 18 months (22). Osteolytic destruction was found in the bone metastases, so we tentatively applied Denosumab to supplement chemotherapy. According to the results of imaging and pathological examination, the bone destruction of the metastases was significantly suppressed and calcified after treatment, and the volume of local metastases was significantly reduced.

Pulmonary metastasis, especially when the metastasis cannot be resected, was the main factor of poor prognosis in patients with osteosarcoma. Anlotinib is a new multi-target tyrosine kinase inhibitor, which has been proven to have good anti-tumor effects on a variety of solid tumors, including non-small cell lung cancer and soft tissue sarcoma, by blocking the phosphorylation of VEGFR2 and PDGFR (18, 28). *In vitro* simulation experiments of human osteosarcoma, it is confirmed that Anlotinib can inhibit the growth of osteosarcoma cells and increase their sensitivity to chemotherapy drugs (29). In the animal experiment of the osteosarcoma transplantation model, it was found that Anlotinib and doxorubicin can significantly reduce the tumor growth rate and reduce tumor volume (30). In addition, when patients with delayed pulmonary metastasis of osteosarcoma are treated with Anlotinib, the tumor progression can be significantly suppressed and the tumor volume is significantly reduced (28).

In this case, after the comprehensive conservative treatment of Denosumab, Anlotinib and DIA chemotherapy, the activity of tumor cells was completely inhibited in the multiple metastases except for in the proximal left femur. Moreover, the multiple bone metastases showed different extent of high-density calcification, and the volume of the metastases in the left fourth rib was significantly reduced. The clinical outcome of Denosumab and Anlotinib in relevant literature is consistent. We believe that the pain relief, suppressed tumor growth, and increased osteogenesis of this patient after treatment is the combined effect of comprehensive treatment. The significant calcification of multiple bone metastasis should be the main role of Denosumab. The inhibition of tumor activity in lung metastasis may be closely related to the use of Anlotinib. After 6 cycles of chemotherapy, Denosumab and Anlotinib still need to be used for a long time. The activity of tumor cells in the proximal left femur has not been completely suppressed, and close follow-up should be carried out. If the condition worsens, resection of the tumor in the proximal left femur and adjustment of the treatment should be carried out. We found that for patients with osteosarcoma and multiple metastases whose imaging was mainly osteolytic destruction, the comprehensive treatment of Denosumab, Anlotinib, and DIA chemotherapy was effective. Up to date, there is no specific targeted drug for osteosarcoma. According to the current research, patients with osteosarcoma whose imaging is mainly osteolytic destruction can be treated with Denosumab. This may provide a new direction for osteolytic osteosarcoma treatment, but its specific mechanism and scope of the application still need further research.

## Data availability statement

The original contributions presented in the study are included in the article/supplementary material, further inquiries can be directed to the corresponding author.

## Ethics statement

This study was performed by the principles of the Declaration of Helsinki. This study has been approved by the 960th Hospital of PLA. Written informed consent to participate in this study was provided by the participants' legal guardian/next of kin. Written informed consent was obtained from the individual(s), and minor(s)' legal guardian/next of kin, for the publication of any potentially identifiable images or data included in this article.

## Author contributions

DJ and JWa made substantial contributions to the acquisition, analysis, interpretation of data, and writing of this manuscript. MX was responsible for the conception and design of the study. QC, JWu, and XY were assistants of the data analysis and English writing of this manuscript. All authors contributed to the article and approved the submitted version.
